# Expression and localization of aging markers in lacrimal gland of chronic graft-versus-host disease

**DOI:** 10.1038/srep02455

**Published:** 2013-08-29

**Authors:** Masataka Kawai, Yoko Ogawa, Shigeto Shimmura, Shigeki Ohta, Takanori Suzuki, Naoshi Kawamura, Masataka Kuwana, Yutaka Kawakami, Kazuo Tsubota

**Affiliations:** 1 Department of Ophthalmology, Keio University, School of Medicine; 2 Division of Cellular Signaling, Institute for Advanced Medical Research, Keio University School of Medicine; 3 Department of Rheumatology, Keio University School of Medicine

## Abstract

Aging is commonly defined as the accumulation of diverse deleterious changes in cells and tissues with advancing age. To investigate whether aging changes are involved in the lacrimal glands of chronic graft-versus-host disease (cGVHD) model mice, we obtained the specimens from cGVHD model mice, untreated aged and young mice, and examined by histopathology, and immunoblotting. Oxidative stress markers, 8-OHdG, 4-HNE, and hexonoyl lesion (HEL), and other aging markers, p16 and p38, were used to assess the samples. The infiltrating mononuclear cells and endothelia of capillaries in the cGVHD and aged mice expressed the oxidative stress markers and other aging markers, but not in the young mice. Histological changes and the expression of aging markers in the samples from cGVHD mice exhibited similar features to those in aging mice. These results suggest that changes that typically appear with advanced age occur earlier in the lives of mice with lacrimal gland cGVHD.

Aging is commonly defined as the accumulation of diverse deleterious changes ocurring in cells and tissues with advancing age that are responsible for increased risk of disease and death^1^. In addition, cellular senescence involves genomic instability and telomere loss, oxidative damage, genetic programming, and cell death^1^. Several theories about aging, including the free radical theory^1^, immunologic theory^2^, inflammation theory^3^, and mitochondrial theory^4^, are commonly accepted. Among these, the free radical theory seems to be the most widely accepted explanation for the primary chemical reactions underlying the aging process^5^.

Hematopoietic stem cell transplantation (HSCT) is an established treatment for many patients with life-threatening hematological diseases^6^,^7^. HSCT has been increasingly used worldwide over the past three decades. Because many recipients of HSCT become long-term survivors, their quality of life and the possibility of late complications after HSCT have become increasingly important^6^,^8^. We previously reported that dry eye after HSCT is a major symptom of chronic ocular graft-versus-host disease (cGVHD)^9^. The patients of cGVHD often show changes in the ophthalmologic areas associated with aging such as poliosis of eyebrows and eyelashes (Supplementary Figure 1). We have observed that such aging-associated changes can progress rapidly in patients with severe cGVHD. In addition, it has been reported that the risk of conjunctival carcinoma is increasing after HSCT patients similar to aging patients^10^. However, there are few published reports on the aging-related changes in cGVHD patients. It is therefore important to confirm the similarity of changes between aging and cGVHD at a histological level.

In this report, we studied the aging-related changes histologically in the lacrimal glands of a murine model of cGVHD. To analyze the aging changes immunohistologically, we used oxidative stress markers and two other well known aging markers, p16 and p38.

## Results

### Histology

**Figure 1:**
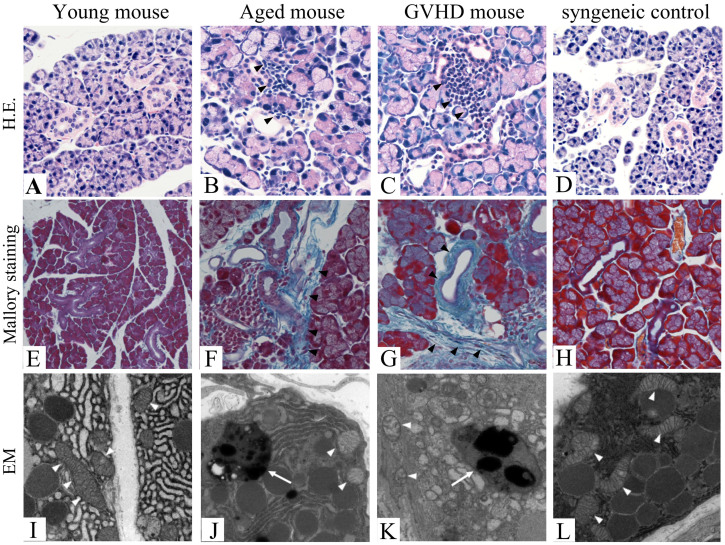
Light microscopic findings and electron microscopic findings of lacrimal glands of aged and cGVHD model mice. Light microscopic findings of the lacrimal gland (A–H). Hematoxylin and Eosin staining of the young mice (A), aged mice (B), cGVHD mice (C), and syngeneic control mice (D). Note that mononuclear cells infiltration is found in the periductal areas of the aged (B, arrowheads) and cGVHD mice (C, arrowheads), whereas only a small number of mononuclear cells are found in the young (A) and syngeneic control mice (D). Mallory staining of the lacrimal gland (E–H). Note that excessive fibrosis (arrowheads) and mononuclear cells infiltration are found in the periductal areas of the aged (F) and cGVHD mice (G) but absent in the young (E) and syngeneic control mice (H). Electron microscopic findings of the lacrimal gland (I–L). Several mitochondria in the aged (J, arrowheads) and cGVHD mice (K, arrowheads) show swollen morphology and disorganization of cristae, whereas those in the young or syngeneic control mice do not show any phenotypic alterations (I, L, arrowheads). Lipofuscin formation is found in the aged (J, arrow) and cGVHD mice (K, arrow) but absent in the young and syngeneic control mice (I, L). H.E.; Hematoxylin and Eosin staining, EM; Electron microscopy. Original magnification. A, D, E, X100, B, C, F–H, X200, I–L, X15000.

Firstly, we examined the histological findings in the lacrimal glands of the aged, cGVHD, and syngeneic control mice and compared them with those in the young mice. In the young and syngeneic control mice, the lacrimal glands are a multilobular tissue composed of acinar, ductal, and myoepithelial cells, with most of the cells being acinar cells. The ducts were orderly lined by uniform epithelia (Figure 1A, D). In the aged mice, epithelia around the ducts were lower than those in the young mice and mononuclear cells were found infiltrating in the periductal areas (Figure 1B). The percentage area of infiltration and total lacrimal gland is 11.0 ± 6.5% (N = 3). Only small numbers of mononuclear cells were found in the young and syngeneic control mice (Figure 1A, D). Cell infiltration and duct abnormalities as seen in the aged mice were also found in the cGVHD mice (Figure 1C). The percentage area of infiltration and total lacrimal gland is 13.8 ± 4.1% (N = 6) in the onset of cGVHD mice at 3 weeks after transplantation. Excessive fibrosis was scarcely detected in the young and syngeneic control mice (Figure 1E, H) but observed in the periductal areas of both the aged (Figure 1F, arrowheads) and cGVHD mice (Figure 1G, arrowheads). These results suggested that histological changes of lacrimal gland from the cGVHD mice are similar to those from the aged mice.

### Electron microscopic findings

To further examine the presence of age-related changes in the lacrimal glands, we analyzed the ultrastructural morphology of the lacrimal gland using transmission electron micrography.

It is generally accepted that lipofuscin play a fundamental role in the aging process, and becomes a focus of aging and stress research. Lipofuscin is a strongly oxidized material composed of altered protein, lipid and even oligosaccharides, in addition to 2% transition metals such as iron, zinc, manganese, and copper^11^. Lipofuscin deposits were found in the cytoplasm of acinar cells in the cGVHD mice (Figure 1K, arrow) as well in the aged mice (Figure 1J, arrow) but scarcely detected in the young or syngeneic control mice (Figure 1I, and L). We also observed certain ultrastructural changes in the mitochondria by transmission electron microscopic examination. Several mitochondria showed swollen morphology and disorganization of cristae in the cGVHD mice (Figure 1K, arrowheads) as well in the aged mice (Figure 1J, arrowheads), whereas the mitochondria did not show any phenotypic alterations in the young or syngeneic control mice (Figure 1I and L). These results indicate that mitochondrial alterations and accumulation of lipofuscin are changes which also occur with aging.

### Immunohistochemistry

To investigate whether cellular aging changes occur in cGVHD tissue, we examined the expression of aging markers in lacrimal gland tissue sections prepared from the young, aged, and cGVHD mice. Cellular senescence was assessed by the expression of 8-OHdG, 4-HNE, and HEL, which are widely used markers for oxidative stress.

**Figure 2:**
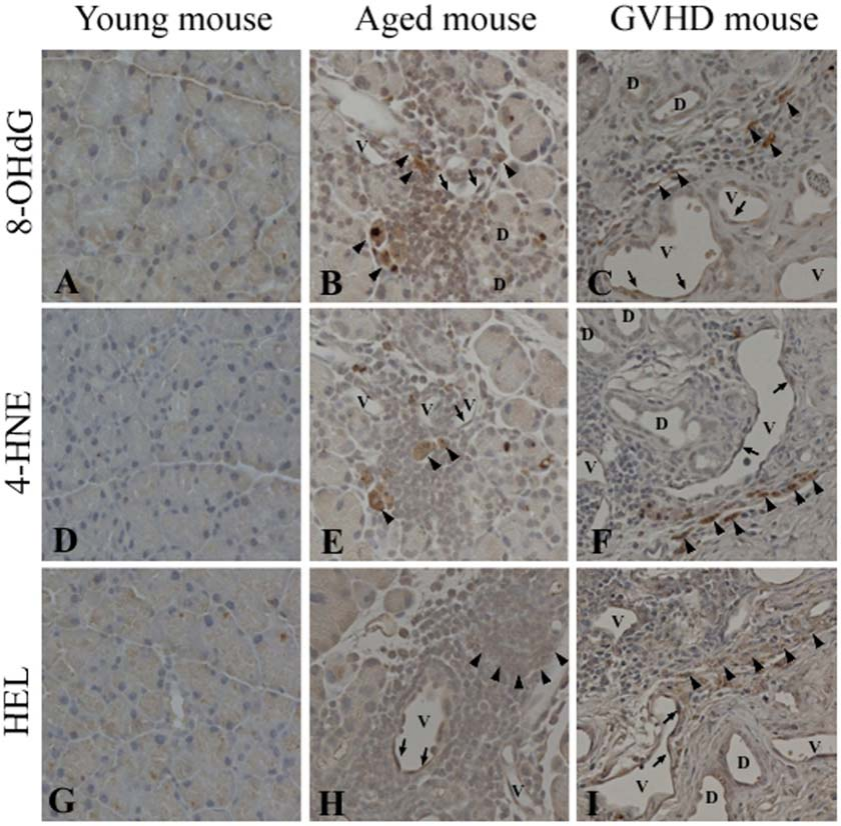
Expression of oxidative-stress markers in the lacrimal glands of aged and cGVHD model mice. Representative lacrimal gland sections immunostained for 8-OHdG (A–C), 4-HNE (D–F), or HEL (G–I). In the aged mice, some mononuclear cells around periductal vessels (arrowheads) and some endothelial cells of blood vessels (arrows) are positive for 8-OHdG (B), 4-HNE (E) or HEL (H), whereas few positive cells are found in the young mice (A, D, G). In the cGVHD mice, some mononuclear cells (arrowheads) and blood vessel endothelial cells (arrows) are positive for oxidative-stress markers (C, F, I) as well as in the aged mice. D; Duct, V; Blood vessel. Original magnification, A, B, D, E, G, H, X400, C, F, I, X200.

8-OHdG is a major oxidative base lesion in DNA or nucleotides, and is induced by reactive oxygen species (ROS)^12^. 8-OHdG-positive cells were scarcely found in the lacrimal glands from the young mice (Figure 2A). In the aged mice, some of the infiltrating mononuclear cells around periductal vessels and endothelia of blood vessels were positive for 8-OHdG (Figure 2B). In the cGVHD mice, some of the infiltrating cells and endothelia of blood vessels expressed 8-OHdG (Figure 2C), as seen in the aged mice.

4-HNE is an indicator of lipid peroxidation and protein damage, and is considered a second toxic messenger of ROS^13^. In the young mice, few 4-HNE-positive cells were found (Figure 2D). In the aged and cGVHD mice, some infiltrating cells and blood-vessel endothelial cells were positive for 4-HNE (Figure 2E, F).

HEL, a lipid hydroperoxide-modified lysine residue, is formed by ROS and is considered a useful biomarker for the initial stage of lipid peroxidation. Few HEL-positive cells were found in the young mice (Figure 2G). In the aged and cGVHD mice, some infiltrating cells and vessel endothelial cells were positive for HEL (Figure 2H, I).

**Figure 3:**
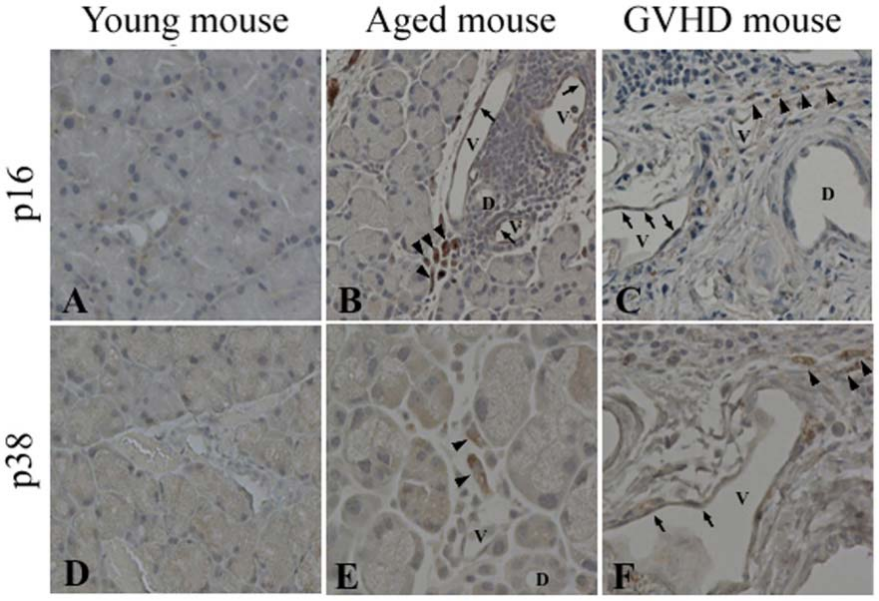
Expression of aging markers in the lacrimal glands of aged and cGVHD model mice. Immunohistochemistry for other aging markers in lacrimal glands. Representative lacrimal gland sections immunostained for p16 (A–C) or p38 (D–F). In the aged (B, E) and cGVHD mice (C, F), some mononuclear cells around periductal vessels (arrowheads) or some endothelial cells of blood vessels (arrows) are positive for p16 or p38 whereas few positive cells are found in the young mice (A, D). D; duct, V; Blood vessels. Original magnification. A–D, F X200, E, X400.

To confirm that aging-associated changes occurred in the cGVHD tissue, we further examined the expression of p38 MAPK (p38) and p16. p38, a member of the MAP kinase family, is activated by multiple environmental stresses^14^ and inflammatory cytokines, and has been shown to be involved in the induction of senescence^15^,^16^. p38 is notoriously associated with inflammatory conditions, including aging, and we tested whether it also is up-regulated in cGVHD. p16, a tumor suppressor molecule, has been identified as one of the major effectors of p38 kinase in the regulation of senescence phenotypes^17^ and is used as a biomarker of aging^18^. p16 or p38 positive cells were scarcely detected in the young mice (Figure 3A, D). In the aged mice, some of the infiltrating mononuclear cells around the periductal vessels expressed p16 or p38 (Figure 3B, E). Some endothelial cells of blood vessels also expressed p16 (Figure 3B). In the cGVHD mice, some of the infiltrating cells around the periductal vessels and endothelial cells of blood vessels were positive for p16 or p38 (Figure 3C, F) as in the aged mice (Figure 3B, E). These results suggested that immune aging and endothelial cellular aging arise in lacrimal gland cGVHD microenvironment.

**Figure 4:**
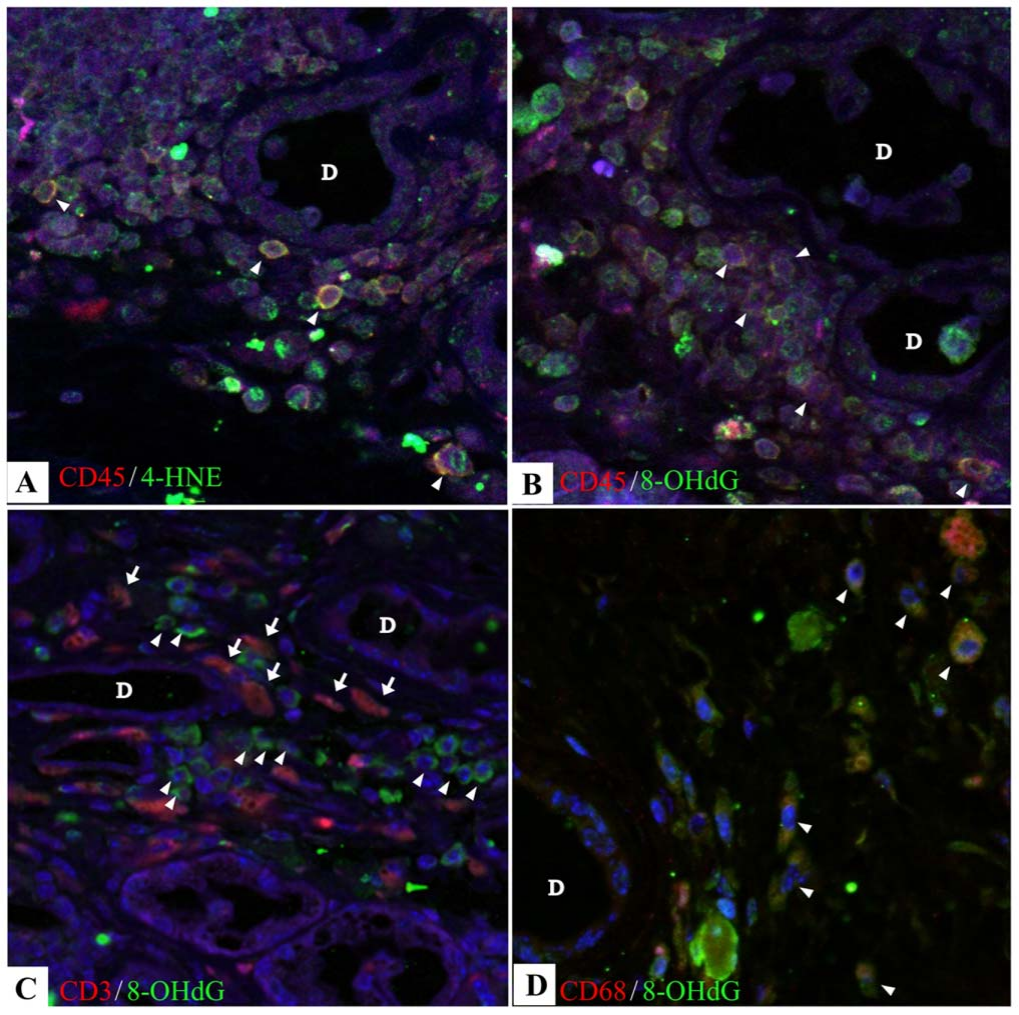
Macrophages are possible cells expressing the oxidative stress markers in the cGVHD model mice. Immunofluorescence double staining of CD45 and 4-HNE (A), CD45 and 8-OHdG (B), CD3 (arrows, red in C) and 8-OHdG (arrowheads, green in C) (C) and CD68 and 8-OHdG (D) on the specimens from animal model of cGVHD. Arrowheads (yellow in A, B, and D); Cells co-expressing a corresponding marker and 8-OHdG. Arrowheads (C): CD3. A, Acini; D, duct. (A–D) Original magnification. X400.

To elucidate the characterization of infiltrating inflammatory cells expressing aging markers, we performed double staining using immunofluoresecein technique. Some inflammatory CD45^+^ leukocytes co-expressed 4-HNE (Figure 4A) and 8-OHdG (Figure 4B). However, CD3^+^ cells, CD11c^+^ cells, and CD19^+^ cells did not co-expressed 4-HNE nor 8-OHdG (Data not shown). CD3^+^ T cells interacted with 8-OHdG^+^ cells, but not co-expressed each other (Figure 4C). Interestingly, CD68^+^ macrophages co-expressed 8-OHdG in the tissue section of the lacrimal gland from the cGVHD mice (Figure 4D).

**Figure 5:**
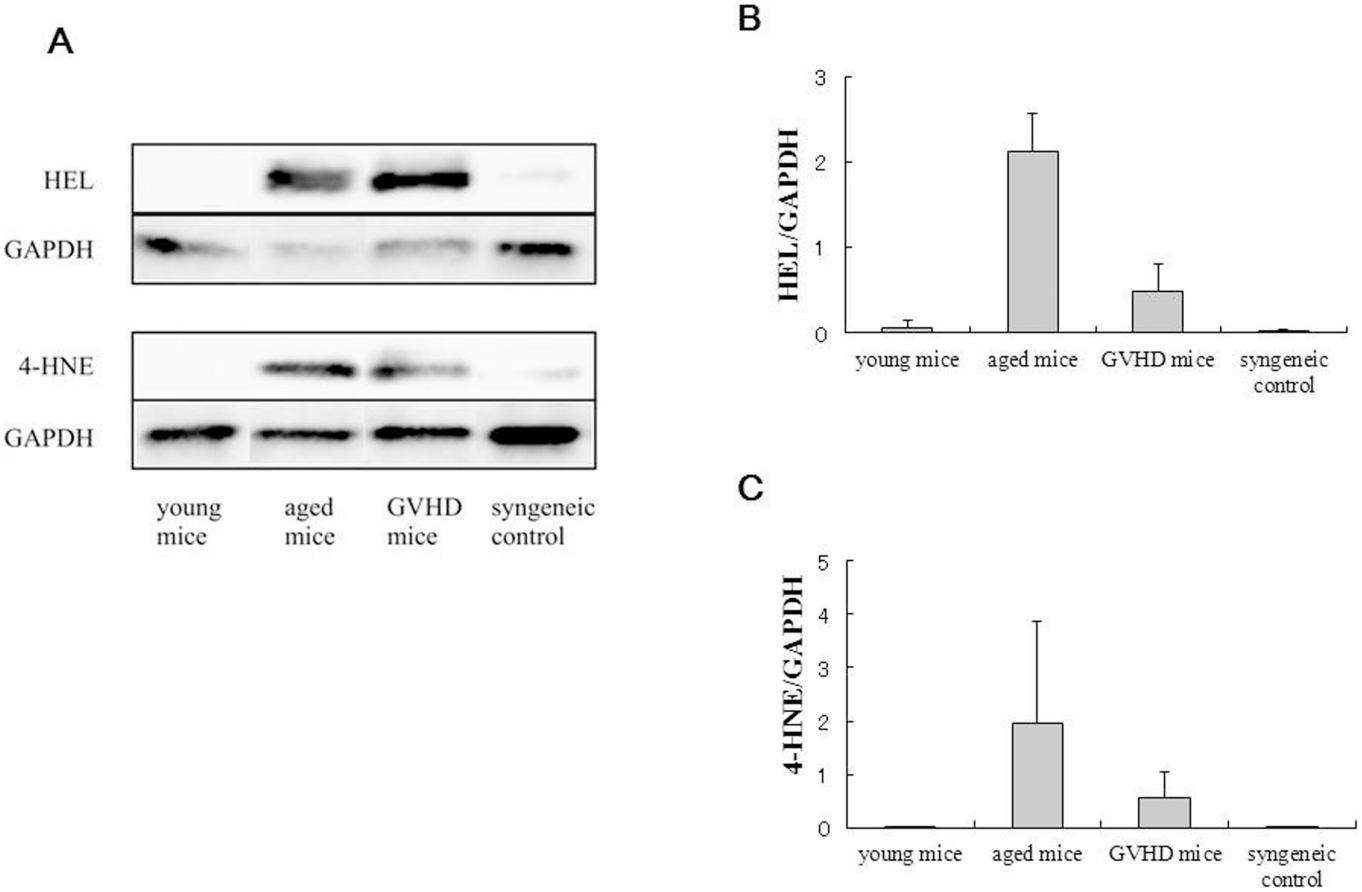
Protein expression of HEL and 4-HNE in the lacrimal glands in the aged and cGVHD model mice. (A) The cropped blots are representative of samples from the young mice (n = 3), aged mice (n = 3), cGVHD mice (n = 3) and syngeneic control (n = 2). The expression of HEL and 4-HNE in the aged and cGVHD mice was notably higher than in the young mice. The full-length gels and blots are shown in the supplementary figure 3. (B, C) The corresponding quantitative data of HEL (B), and 4-HNE(C), normalized to the internal control, GAPDH, are shown. Bars indicate standard deviation.

Western blot analysis showed that the expression of HEL and 4-HNE in the aged mice was notably higher than in the young mice and that those markers were also expressed in the cGVHD mice. The results of western blot analysis confirmed immunohistochemical findings showing enhanced expression of oxidative stress markers in the aged and cGVHD mice (Figure 5A–C, Supplementary Figure 3).

## Discussion

BMT recipients, especially those with cGVHD, are known to be at a significantly higher risk for new solid cancers than the general population^19^. Some cases of conjunctival carcinoma that may be related to cGVHD have been reported^10^. The high risk of cancer development in cGVHD patients may be related to cellular senescence. In fact, we often observe aging-associated changes during ophthalmic examinations after HSCT, such as poliosis of the eyebrows and eyelashes or skin wrinkles, in patients with cGVHD (Supplementary Figure 1). Moreover, in a preliminary experiment, we found p16 positive cells in lacrimal gland specimen from patient with cGVHD whereas scarcely found in that with Sjögren syndrome (Supplementary figure 2). Such clinical observations and preliminary histological findings led us to hypothesize that histological changes in cGVHD may be similar to those occurring in aging. Here, to prove this hypothesis, we compared the lacrimal glands of a murine model of cGVHD with those of aged mice histologically.

Inflammatory cell infiltration, fibrosis in the periductal area, and duct pathology were found in this murine model of cGVHD as well as in aged mice. In humans, the incidence of inflammatory cell infiltration in lacrimal glands is known to increase with age^20^,^21^, as in multiple other organs^22^. Fibrosis and duct pathology have been also observed in the lacrimal glands of aged humans^20^,^21^. We previously reported similar changes occurred in the lacrimal glands of patients with cGVHD^23^. Thus, the histological changes observed in cGVHD model mice also occur in human cGVHD and those changes are found in aging.

Electron microscopic findings further suggest the similarity between changes in cGVHD and those in aging. Mitochondrial alterations with aging such as swelling or loss of cristae have been well known^24^,^25^. We found such changes not only in the aging mice but in the cGVHD mice. Lipofuscin deposits are generally accepted as changes occurred with aging in humans and have been reported to occur in murine lacrimal glands with aging^26^. We also found lipofuscin deposits in the cGVHD mice. The accumulation of lipofuscin-like inclusions as a result of an increased oxidative stress in the lacrimal gland of cGVHD might have been influenced by accelerated aging process. In addition, the contributions of lipofuscin accumulation to cumulative oxidative damage to the acinar cells may be involved in one of the causes of cGVHD related dry eye similar to aging related dry eye. The mechanisms underlying this condition need to be clarified in further investigations.

Some infiltrating cells were positive for oxidative stress markers in both the cGVHD mice and the aged mice. The cGVHD mice received total body irradiation (TBI) in the BMT protocol. Since TBI is known to induce lipid peroxidation in tissues^27^, the findings in the cGVHD mice in this study might have been influenced by TBI. However, neither HEL nor 4-HNE was detected in the syngeneic control mice that were irradiated by same protocol as the cGVHD mice. Furthermore, we confirmed that oxidative stress markers elevated in lacrimal gland tissue in both cGVHD and aged mice, but not in syngeneic control and young mice. Therefore, the radiation level used for this murine model of cGVHD is considered to be relatively low.

Some infiltrating cells in the cGVHD mice or aging mice expressed oxidative stress markers and aging markers whereas acinar cells nor ductal cells scarcely expressed those markers. It is interesting that most of the accumulation which is products of oxidative damage is in infiltrating immune cells, rather than parenchymal cell. In cGVHD group, the time we analyzed was consistent with the onset of cGVHD. There is a possibility the oxidative damage of immune cells might be the earliest sign of immune aging. Recently, macrophages are reported to play a causative role for aging diseases such as atherosclerosis, cancer, and age-related macular degeneration^28^. Other studies have revealed that oxidative stress in mitochondria can activate ROS, leading to inflammasome activation, cytokine production such as IL-1, and IL-18, leading to inflammation and aging^29^,^30^. Our results have suggested that macrophages play some role for generating oxidative stress, and inflammasome activation, followed by cytokine production, leading to parenchymal cell destruction, similar to the process of the development of aging diseases.

It has been proposed that HSCT itself may accelerate aging of the immune system, because long-term engraftment imposes replicative stress on hematopoietic stem cells^31^. In the current study, control mice that received syngeneic BMT did not show the similar changes as seen in the cGVHD mice. It is probably because the examination was carried out in a relatively short period after BMT. Infiltrating cells in the cGVHD mice might express those markers possibly owing to double stress, HSCT and cGVHD. In spite of short period after BMT, the changes of inflammatory events started before the onset of cGVHD in an animal model. Many donor or recipients' cells are reported to migrate into the target organs through blood vessels^32^. However, the interpretation should be careful to explain this observation because the direct evidence is not obtained.

Endothelial cells of blood vessels were also positive for aging markers such as p16, and p38. Vessel endothelia are the first recipient cells encountered by circulating alloreactive donor or cytotoxic recipient T cells after allogeneic HSCT. In addition, vascular injury has been reported in patients with cGVHD^33^. We previously reported that the lacrimal glands of patients with cGVHD contain blood vessels with multilayered basal laminae, which are considered to be a result of repeated damage and repair of vessel walls^23^. It is plausible that repeated damage around the onset of cGVHD induced by the transmigration of abnormal blood cells might cause the aging of vessel endothelia. However, we should be careful to interpret these findings. Further investigation will be needed to prove this phenomenon.

Our findings suggest that histological changes arising in cGVHD share properties with aging-associated changes. What we have found are signs of the accumulation of products that accumulate with aging and oxidative stress in cGVHD mice. Aging is commonly defined as the accumulation of diverse deleterious changes occurring in cells and tissues with advancing age^1^ and it consists of various changes. Therefore, we can not regard changes in cGVHD same as those in aging only by the findings of current study. Further studies investigating the relationship between cGVHD and aging will be useful for clarifying the mechanisms of both cGVHD and the aging process.

## Methods

### Mice

All the experimental procedures and protocols were approved by the ethics committee of Keio University and were in accordance with the Guide for the Care and Use of Laboratory Animals (protocol # 09152).

### Bone marrow transplantation

The murine model of cGVHD was induced by bone marrow transplantation (BMT) according to an established protocol^33^. Typically, 7–8-week-old male B10.D2 (H-2^d^) and female BALB/c (H-2^d^) (Sankyo Laboratory, Tokyo, Japan) mice were used as donors and recipients, respectively, for BMT to produce cGVHD. A standard method using added spleen cells as a source of mature T cells was used. Briefly, recipients were lethally irradiated with 700 cGy from a Gammacel 137 Cs source (Hitachi Medico, LTd, Tokyo, Japan). Donor bone marrow (1 × 10^6^/mouse) and spleen cells (2 × 10^6^/mouse) suspended in RPMI-1640 medium (Life Technologies Japan Ltd, Tokyo, Japan) were then injected into the irradiated mice via the tail vein. A control group of female BALB/c recipient mice received male BALB/c spleen and bone marrow (syngeneic BMT, referred to as control animals). The transplant-receiving animals were maintained in sterile microisolator cages and given autoclaved food and acidified water. The cGVHD or control animals were used for experiments at 3 weeks after transplantation.

### Histology and immunohistochemistry

Three to five animals per group (young mice, aged mice, cGVHD model mice and syngeneic control mice) were studied in each experiment. Three weeks after BMT, the lacrimal glands of the cGVHD mice and sygeneic control mice were removed. The lacrimal glands were also removed from non-treated aged mice (102-week-old BALB/c mice) and young mice (7–8-week-old BALB/c mice). Sections were prepared from formalin-fixed paraffin-embedded tissues and some were used for Hematoxylin and Eosin staining and Mallory staining. For Mallory staining, sections were deparaffinized and rehydrated and stained with 0.1% acid fuchsin for 5 minutes followed by 1% phosphomolybdic acid solution for 15 minutes, and then stained with aniline blue-orange G mixed solution for 10 min. After each staining, sections were washed by distilled water 3 times. For immunohistochemical analyses, sections were deparaffinized. The antigenic epitopes were unmasked using the antigen retrieval method. Then, the sections were soaked in 0.3% hydrogen peroxide in methanol for 30 min at room temperature to inactivate the endogenous peroxidase activity. The sections were then blocked with 10% goat serum for 30 minutes, and incubated overnight at 4°C with the primary antibodies, which were against the oxidative stress markers 8-hydroxy-2′-deoxyguanosine (8-OHdG), 4-hydroxy-2-nonenal histidine (4-HNE), and hexanoyl lysine (HEL), and other aging markers p16 and p38 (Table 1). The sections were then incubated with a peroxidase-labeled anti–rabbit or anti–mouse secondary antibody (Histofine Simple Stain Max PO; Nichirei, Tokyo, Japan) for 45 min at room temperature. The bound antibody was visualized with 3, 3-diaminobenzidine tetrahydrochloride, and cell nuclei were counterstained with hematoxylin for 1 second, then rinsed in water. All the incubation steps were performed in a moist chamber. Briefly, for staining, the tissue sections were treated in a microwave oven for 10 minutes. Images were photographed with a microscope (Nikon COOLSCOPE II, Nikon Corporation, Tokyo, Japan). All the sections were reviewed twice in a blind manner by two independent ophthalmologists (M.K. and Y.O.), to assess the lacrimal gland histological architecture and staining. In some experiments, formalin-fixed paraffin embedded tissue sections were double-stained with a phycoerythrin-conjugated rat anti- mouse CD45 (e-Bioscience, San Diego, CA) on panleukocytes, CD3 (e-Bioscience) on T cells, CD19 (e-Bioscience) on B cells, CD68 (DS Pharma Biomedical, Osaka, Japan) on macrophages or HSP47(Bioss, Boston, MA) on stromal fibroblasts with a rat anti CD45, hamster anti CD3, rat-anti CD11c, rat-anti CD19, rat anti CD68 or rabbit anti HP47 antibody in combination with an Alexa 568 -conjugated goat anti-rat, rabbit or hamster secondary antibody (Life Technologies/Molecular Probe, USA) and double stained with mouse anti 8-OHdG (Japan Institute for the Control of Aging, Shizuoka, Japan) in combination with an Alexa 488-conjugated goat anti mouse secondary antibody (Molecular Probe) (Table 1). Isotype-matched each antibodies were used in control experiments. These sections were mounted and examined with a confocal microscope (LSM700-ZEN 2009; Carl-Zeiss, Göttingen, Germany). At least 3 micrographs were evaluated for each staining by two independent observers (YO, MK).

### Transmission electron microscopic examination

Transmission electron microscopic analysis was performed according to standard protocols^34^. Lacrimal gland specimens were immediately fixed with 2.5% glutaraldehyde in 0.1 M phosphate buffer (pH 7.4) for 4 hours at 4°C and washed three times with 0.1 M phosphate buffer. The samples were then postfixed in 2% osmium tetroxide, dehydrated in a series of ethanol and propylene oxide, and embedded in epoxy resin. One micrometer sections were stained with methylene blue, and the portions containing lacrimal gland structure were thin-sectioned. The sections were collected on mesh grids stained with uranylacetate and lead citrate, and examined under an electron microscope (1230 EXII; JOEL, Tokyo, Japan). All photographs were taken with a bio scan camera (Gatan bio scan camera model 792, Tokyo, Japan).

### Western blot analysis

Lacrimal glands from young, aged, and cGVHD mice were solubilized in 1 ml buffer containing 100 mM 2-amino-2-hydroxymethyl- propane-1,3-diol (Tris; pH 7.5), 10 mM sodium pyrophosphate, 100 mM sodium fluoride, 10 mM EDTA, 10 mM sodium vanadate, 2 mM phenylmethylsulfonyl fluoride, and 1% Triton-X 100 and homogenized using a Polytron PT 1200C homogenizer (Brinkmann Instruments, Westbury, NY). The extracts were then centrifuged at 40,000 × g at 4°C for 5 min to remove insoluble material. Protein concentrations of each sample were determined using a Bio-Rad protein assay kit (Bio-Rad Laboratories, Hercules, CA) with bovine serum albumin as a standard. Identical amounts of proteins were electrophoresed in 10% SDS-PAGE gels and transferred to a nitrocellulose membrane. The membranes were blocked with 5% skim milk powder (Wako, Osaka, Japan) in TBST (20 mM Tris-HCl, 150 mM NaCl, and 0.02% Tween-20, pH 7.4) for one hour at room temperature, then incubated with anti-4-HNE (1:26), HEL (1:50) antibodies overnight at 4°C. After three washes in TBST, the blots were incubated with the appropriate secondary antibodies conjugated with horseradish peroxidase (1:4000, anti-mouse; GE Healthcare, Waukesha, WI) (1:2000, anti-rabbit; GE Healthcare) for 1 hr at room temperature. Signals were detected with ECL Prime Western Blotting Detection Reagent (GE Healthcare) and exposed to Hyperfilm (GE Healthcare).

As a control for equal protein loading, blots were restained using anti-actin (1:4000; Sigma-Aldrich, St. Louis, MO) and GAPDH (1:200; Santa Cruz Biotechnology, Inc., Santa Cruz, CA) antibodies.

## Author Contributions

M.K. and Y.O. wrote the main manuscript text and M. K. and Y.O. prepared figures 1–5 and Supplementary figure 1–2. M.K. and Y.O. conducted most of experiments. S.O., T.S. and N.K. assisted some of the experiments and analyzed data. S.S. and K.T. organized and supervised this study. Y.K. and M.Kuwana advised the selection of the mouse model and made a critical comment for this study. All authors reviewed the manuscript.

## Supplementary Material

Supplementary Information Kawai, M, et al. Supplementary Methods

## Figures and Tables

**Table 1:** Antibodies Used for Immunohistochemistry

Antibody	Dilution	Clone	Commercial Source
8-OHdG	×10	N45.1	Japan Institute for the Control of Aging, Shizuoka, Japan
4-HNE	×4	HNEJ-2	Japan Institute for the Control of Aging, Shizuoka, Japan
HEL	×10	5H4	Japan Institute for the Control of Aging, Shizuoka, Japan
p16	×100	M-156	Santa Cruz Biotechnology Inc., Santa Cruz, CA
p38	×40	12F8	Cell Signaling Technology, Beverly, MA
CD45	×100	30-F11	eBioscience, San Diego, CA
CD3e	×100	145-2C11	eBioscience, San Diego, CA
CD11c	×100	N418	eBioscience, San Diego, CA
CD19	×100	eBio1D3(1D3)	eBioscience, San Diego, CA
CD68	×100	FA-11	DS Pharma Biomedical, Osaka, Japan
HSP47	×100		Bioss, Boston, MA

8OHdG; 8-hydroxy-2′-deoxyguanosine, 4HNE; 4-hydroxy-2-nonenal histidine, HEL; hexanoyl lysine, HSP47; heat shock protein 47.
