# Supplementary material for: Expression and localization of aging markers in lacrimal gland of chronic graft-versus-host disease
Source: Sci Rep. 2013 Aug 29;3:2455. doi: 10.1038/srep02455 (PMC3756340; doi:10.1038/srep02455)
Supplement: Supplementary Information — Kawai, M, et al. Supplementary Methods [file srep02455-s1.pdf]

Title; Expression and localization of aging markers in lacrimal gland of chronic graft-versus-host disease

Authors; Masataka Kawai, Yoko Ogawa, Shigeto Shimmura, Shigeki Ohta, Takanori Suzuki, Naoshi Kawamura, Masataka Kuwana, Yutaka Kawakami, and Kazuo Tsubota

Supplementary methods

Lacrimal gland specimens of human lacrimal glands were obtained by biopsy for the diagnosis of chronic graft-versus-host disease and Sjögren’s syndrome. Written informed consent was obtained from all patients. Lacrimal gland biopsy specimens were examined histologically using conventional techniques and immunohistochemistry. All the research and measurements for human subjects followed the tenets of the Declaration of Helsinki. This study was also approved by the ethics committee of the Keio University School of Medicine. P16 staining on paraffin sections was performed as described in p16 staining on mouse lacrimal gland paraffin sections.

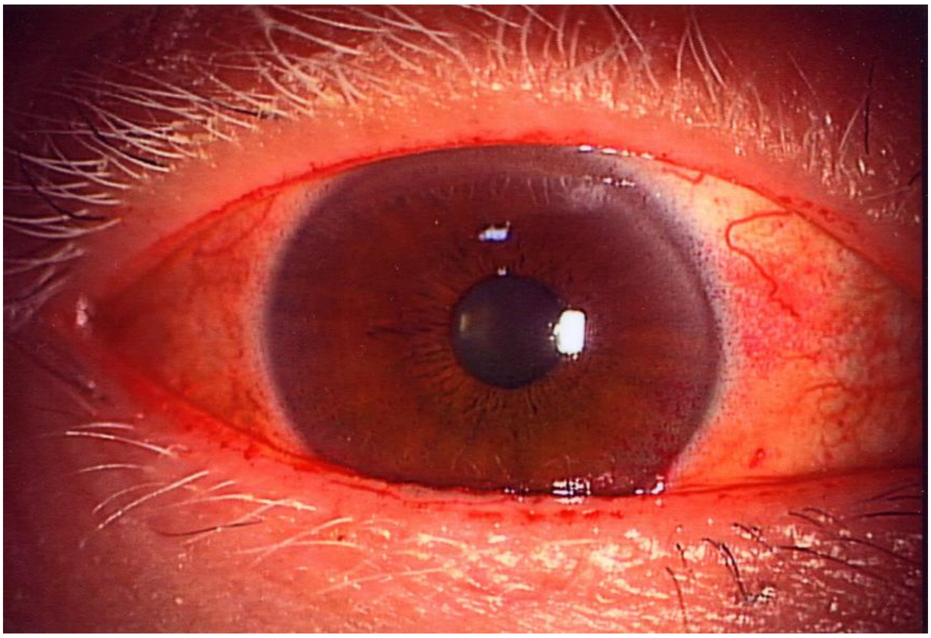

Supplementary figure 1. Kawai, M et al.

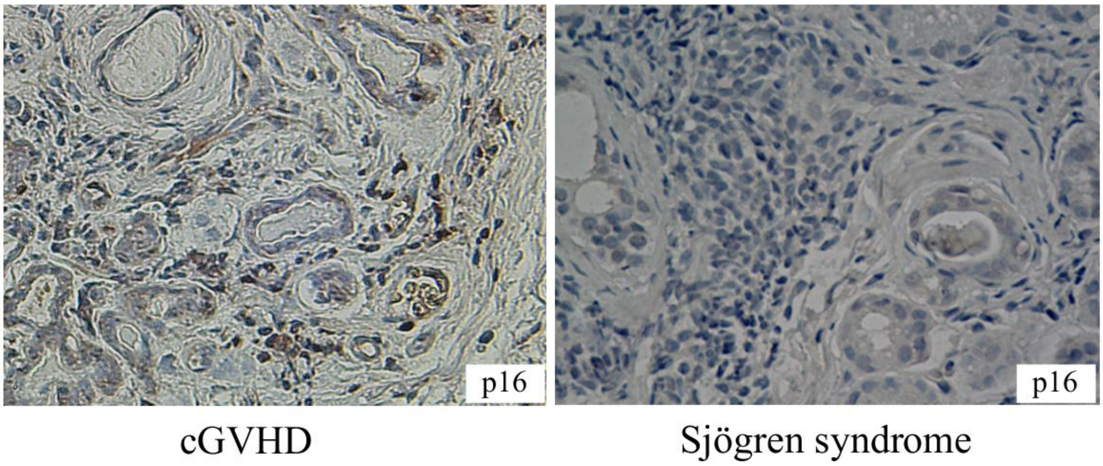

Supplementary figure 2. Kawai, M, et al.

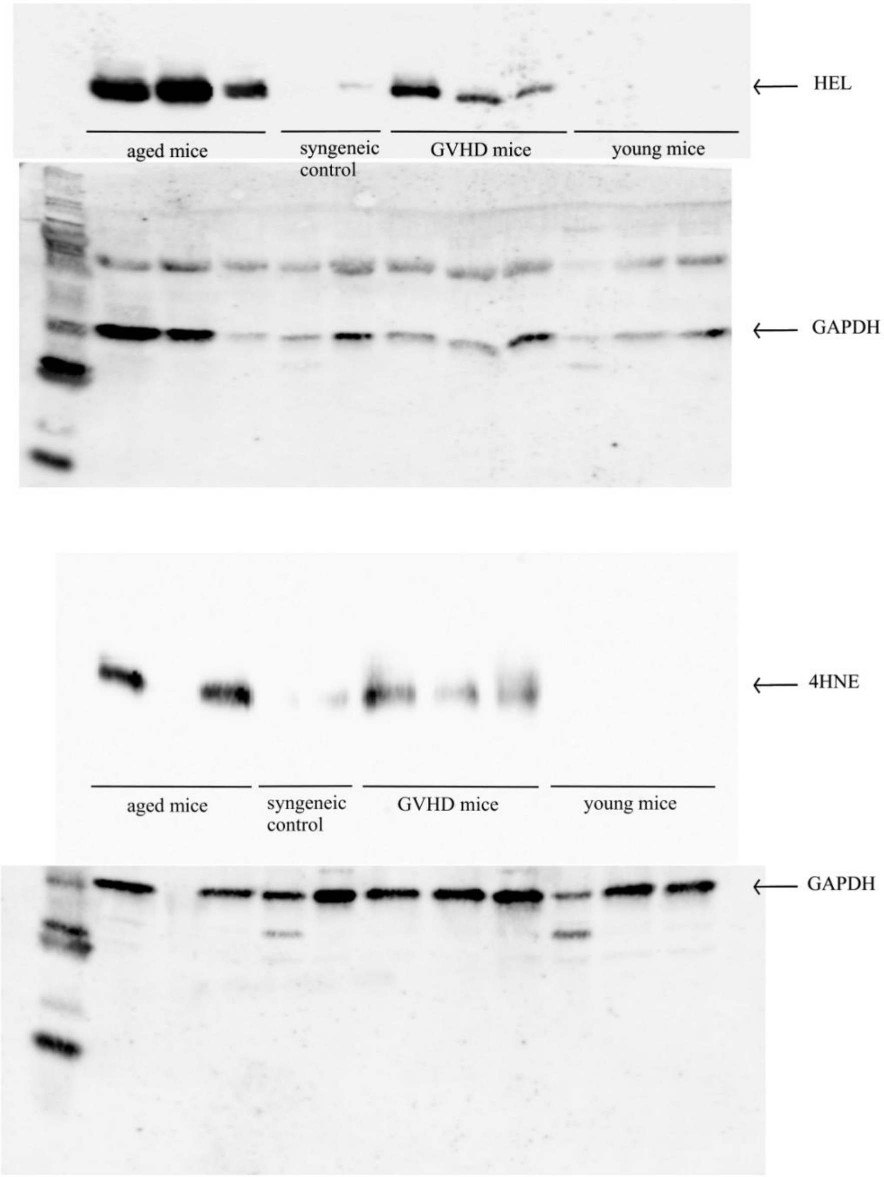

Supplementary figure 3, Kawai M, et al.

Supplementary figure legends

Supplementary figure 1. Poliosis in a patient with cGVHD. Note extensive poliosis in a patient with cGVHD shortly after the onset of cGVHD related dry eye (a 60 year-old female). Informed consent was obtained from the patient.

Supplementary figure 2. Representative images of p16 expression and localization on lacrimal glands from a cGVHD related dry eye patient at 10 months after allogeneic bone marrow transplantation (BMT). (a 53 years old male who received unrelated BMT for acute lymphocytic leukemia.) Note $p16^+$ cells infiltrate around ducts and vessels and expressed on some of blood vessels similar to animal model of cGVHD (left) compared with that from a Sjogren’s syndrome patient (a 60 years-old female). D; duct, V; Blood vessels. Original magnification. X200.

Supplementary figure 3. Western blot analysis of HEL and 4HNE. The full length blots of samples from the young mice (n=3), aged mice (n=3), cGVHD mice (n=3) and syngeneic control (n=2). The expression of HEL and 4HNE in the aged and cGVHD mice was notably higher than in the young mice.
